# Unusual presentation of recurrent follicular lymphoma as diffuse granular shadow

**DOI:** 10.1002/rcr2.710

**Published:** 2021-02-22

**Authors:** Takayasu Ito, Shotaro Okachi, Yuichi Ishikawa, Satoko Shimada, Keiko Wakahara, Naozumi Hashimoto

**Affiliations:** ^1^ Department of Respiratory Medicine Nagoya University Graduate School of Medicine Nagoya Japan; ^2^ Department of Hematology and Oncology Nagoya University Graduate School of Medicine Nagoya Japan; ^3^ Department of Pathology and Laboratory Medicine Nagoya University Hospital Nagoya Japan

**Keywords:** Follicular lymphoma, granular shadow, transbronchial lung biopsy

## Abstract

A 75‐year‐old man was diagnosed with advanced follicular lymphoma because of enlarged cervical lymph nodes. He received chemotherapy and was in complete remission for four years. However, after four years, he developed diffuse lymphadenopathy in the abdominal and iliac area suspected to be recurrent follicular lymphoma. At the time, he was asymptomatic and did not have any accompanying lung lesions. Due to his asymptomatic state, careful monitoring was chosen. Later, he developed diffuse granular shadow in the lung fields. A definite diagnosis was difficult to achieve without histological findings. Therefore, transbronchial lung biopsy of the lesions was performed. The pathology and immunohistochemistry of the lesions revealed recurrent follicular lymphoma. Although the frequency of recurrent follicular lymphoma presenting with diffuse granular shadow is uncommon, recurrent malignant lymphoma should be considered as a differential diagnosis in case with a history of malignant lymphoma.

## Graphical abstract

We herein describe a case of pulmonary lesion presenting with diffuse granular shadow that was diagnosed as recurrent follicular lymphoma.

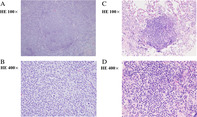

## Introduction

Follicular lymphoma is an indolent B‐cell lymphoproliferative disorder of transformed follicular centre B‐cells. A past report showed that the recurrence of follicular lymphoma involves diffuse lymphadenopathy and bone marrow invasion; however, it is uncommon for other healthy organs to be involved [1]. In a review by Lee et al., the most frequent chest radiography finding in patients with malignant lymphoma was multiple pulmonary nodules, whereas malignant lymphoma presenting with diffuse granular shadow was less frequent (6%) [2]. The main differential diagnoses considered for patients with malignant lymphoma who have diffuse granular shadow on chest computed tomography (CT) are an infectious disease, fungal disease, miliary tuberculosis, and metastatic lung cancer. However, in patients with a history of malignant lymphoma, diffuse granular shadow should also be considered as a sign of malignant lymphoma relapse, as this can happen on rare occasions. We herein described a case of pulmonary lesion presenting with diffuse granular shadow that was diagnosed as recurrent follicular lymphoma.

## Case Report

**Figure 1:**
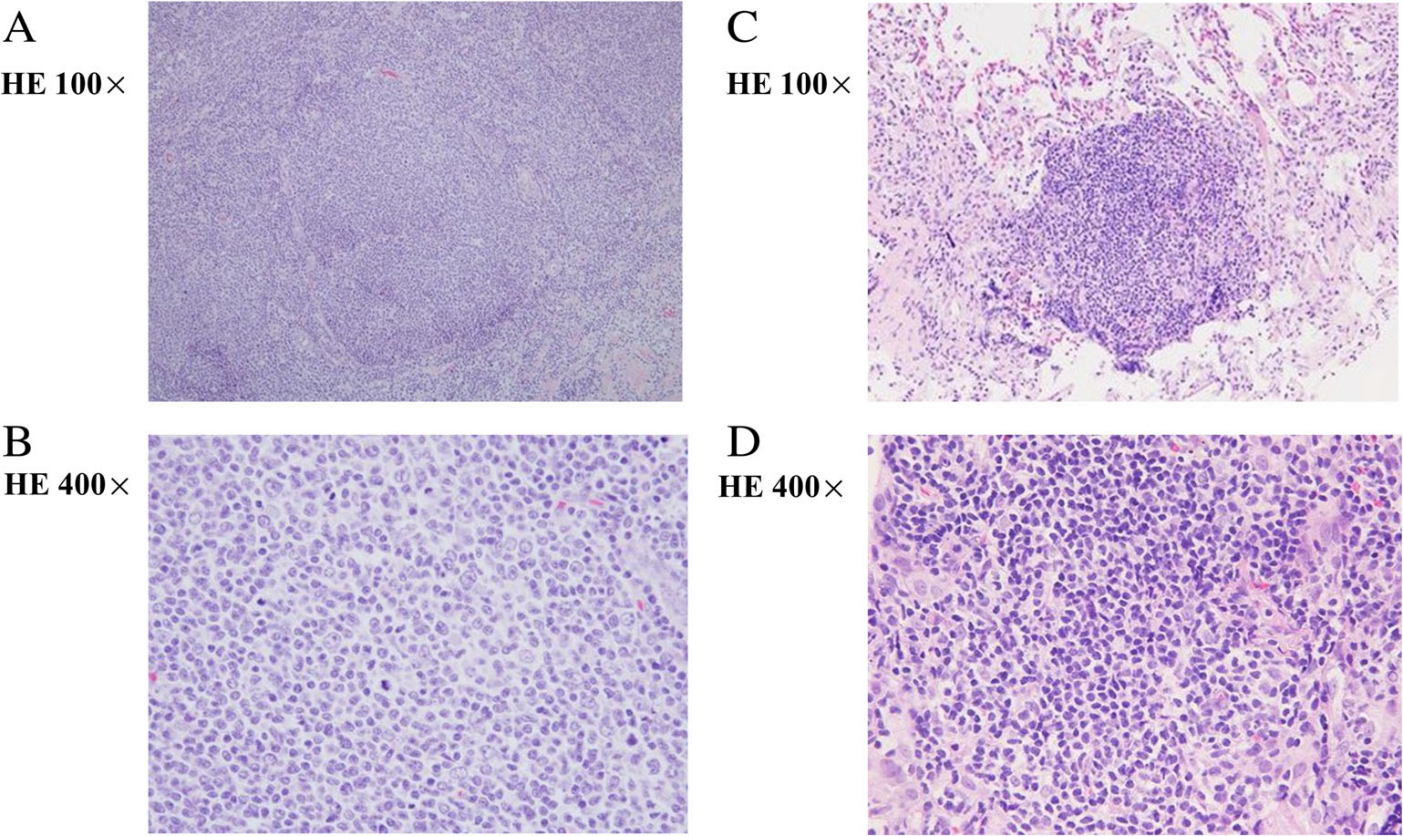
Histopathological findings from cervical lymph nodes specimens (A, B) and those obtained by transbronchial lung biopsy (C, D). (A, B) Histopathological findings showed a mixture of cleaved centrocytes and non‐cleaved centroblasts in the high‐powered view, forming a nodular pattern in the low‐powered view (haematoxylin and eosin stain). (C, D) Low‐ and high‐powered view of the accumulated small centrocyte‐like cells (haematoxylin and eosin stain).

**Figure 2:**
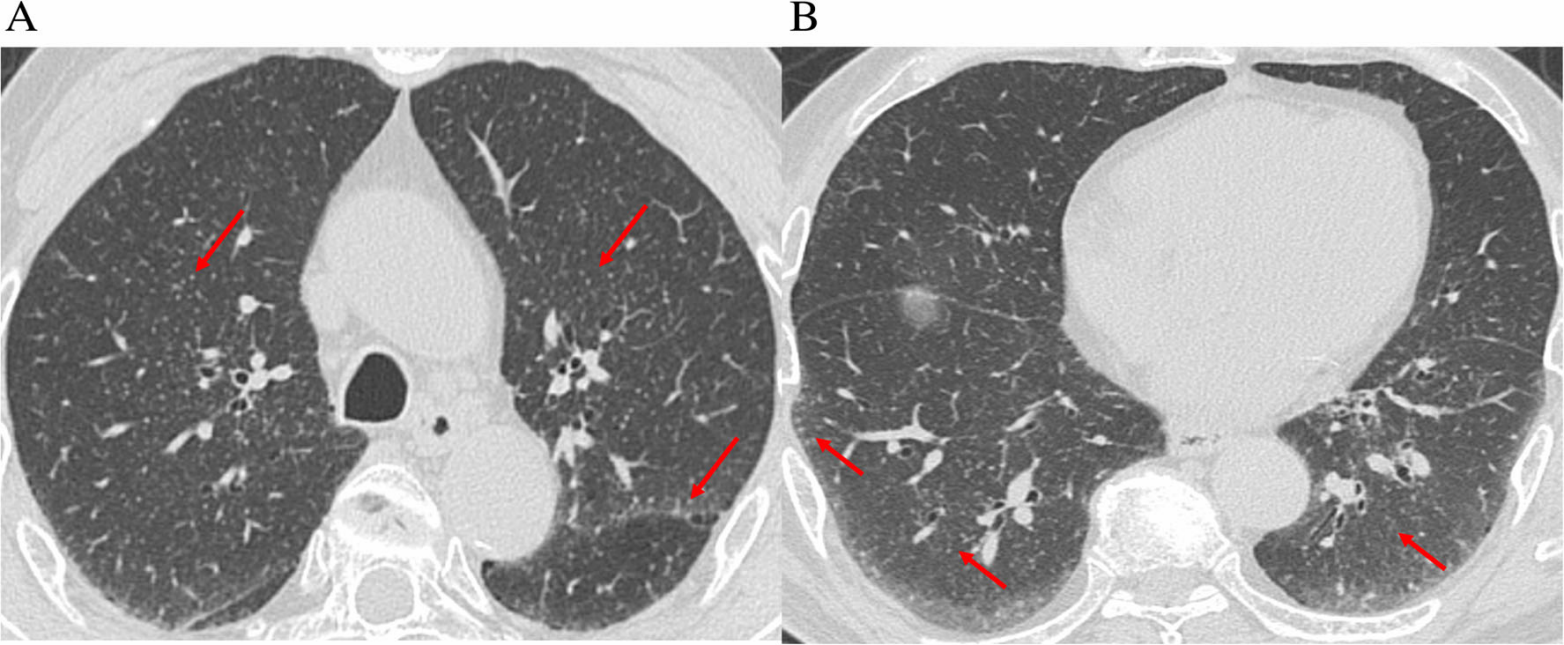
Chest computed tomography (CT). (A, B) CT reveals diffuse granular shadow along with the interlobular pleura and bronchial vessel bundles in the lung fields.

A 75‐year‐old man had enlarged left cervical lymph nodes without lung lesions five years previously. The antibiotic therapy with amoxicillin hydrate was not effective against the lesions. Therefore, a biopsy of the lesions was later performed. The pathological finding was follicular lymphoma grade 3a (Fig. 1A). CT, fluorodeoxyglucose‐positron emission tomography, and bone marrow aspiration revealed the follicular lymphoma was clinical stage IV. Following this diagnosis, the medication R‐THP‐COP consisting of rituximab, pirarubicin, cyclophosphamide, vincristine, and prednisolone was administered. After eight months, the patient was in complete remission. Abdominal and iliac CT showed diffuse lymphadenopathy in the external iliac, inguinal, and paraaortic area over four years after the patient's initial treatment. We considered these lesions to be recurrent follicular lymphoma. Because the patient was in asymptomatic state, he was carefully monitored. Later, new small granular lesions appeared as diffuse pattern on the patient's chest CT (Fig. 2). Moreover, the small granular lesions were observed along with the interlobular pleura and bronchial vessel bundles. The differential diagnoses considered were fungal disease, miliary tuberculosis, sarcoidosis, and lymphoproliferative disorder such as recurrent follicular lymphoma. Lactate dehydrogenase and interleukin‐2 receptor levels were 193 IU/mL (normal range: 120–242 IU/mL) and 942 IU/mL (normal range: 190–650 IU/mL), respectively. Smear and culture microscopic examinations were negative for acid‐fast bacteria in the sputum. Moreover, results of an interferon‐gamma release assay and a β‐d glucan assay were negative. To diagnose the lesions, we performed transbronchial lung biopsy (TBLB) in the right B8, B9, and B10 regions. The pathological findings from the right B8 area showed a cluster of small lymphocytes cells (Fig. 1B). The immunohistochemistry of these cells was positive for CD20, CD10, and bcl‐2, but negative for CD3, CD5, and cyclin D1. The lung lesion was considered to be recurrent follicular lymphoma. As the patient was in an asymptomatic state and did not desire treatment, we decided to monitor him carefully rather than initiate treatment. Moreover, one year later, the lung lesions and diffuse lymphadenopathy in the external iliac, inguinal, and paraaortic area remained, but were in a stable state.

## Discussion

We report on a patient who presented with diffuse granular shadow as a sign of recurrent follicular lymphoma. Involvement of the lung at initial diagnosis was noted in 4% of patients with non‐Hodgkin lymphoma. Moreover, pulmonary involvement is rarely noted in the recurrent stage of non‐Hodgkin lymphoma [2]. Recurrent or secondary pulmonary lymphoma shows various patterns on chest CT [3]. Multiple pulmonary nodules are the most common CT findings of recurrent lymphoma of the lung [4]. The least common presentation of recurrent lymphoma is miliary or haematogeneous patterns [5].

Follicular lymphoma is often accompanied by diffuse lymphadenopathy and bone marrow invasion. However, recurrent follicular lymphoma rarely affects other organs including the lungs [1]. There are little reports regarding pulmonary follicular lymphoma. Terada reported a case of primary pulmonary follicular lymphoma with a small (15 mm in the largest diameter) nodule in the right middle lobe [6]. Recently, Katano et al. reported the first case of pulmonary follicular lymphoma showing diffuse micronodules with a perilymphatic distribution on high‐resolution CT [7]. Thus, we encountered a rare case of recurrent follicular lymphoma in a patient presenting with diffuse granular shadow, observed along with the interlobular pleura and bronchial vessel bundles on chest CT. In our TBLB samples, follicular lymphoma was diagnosed only in one small lesion located in the interstitial space near the terminal bronchioles and bronchovascular bundle. However, it is difficult to evaluate pathologically, which corresponds to the radiological findings, because of the small‐sized specimen obtained by TBLB.

Without histological findings, we could not diagnose whether the lesion was a recurrent follicular lymphoma, infectious disease, or another type of lesion. Bronchoscopy techniques including TBLB or transbronchial cryobiopsy (TCB) and surgical lung biopsy (SLB) were used for the histological diagnosis of pulmonary lesions. SLB is the most accurate modality for the histological analysis and immunostaining of a larger amount of materials, but it requires general anaesthesia and is therefore invasive when used as the first diagnostic procedure. Although it may be difficult to diagnose malignant lymphoma based on TBLB, miliary tuberculosis and sarcoidosis are diagnosed by TBLB, and we first performed TBLB. In recent years, TCB, when compared to TBLB, has been used as a bronchoscopic procedure for pulmonary lesions associated with malignant lymphoma [8]. In our case, TCB might be also useful because of the collection of larger and less crushed samples. It was important to inform the pathologists that we suspected the diffuse granular shadow to be a recurrent follicular lymphoma, which has no typical radiographic pattern to establish a definite diagnosis using a small‐sized specimen. For the treatment of recurrent lymphoma, it is essential to compare the histological grade at recurrent diagnosis with that at first diagnosis. In our case, the histological grade of the follicular lymphoma at first diagnosis and at recurrent diagnosis was 3a and 1, respectively. Moreover, microscopic findings of a 0.4‐mm nodular lesion were compatible with the diffuse granular pattern noted on chest CT. The chest CT and pathological findings reflected the aspect of low‐grade lymphoma even in recurrent stage.

Patients with asymptomatic follicular lymphoma do not require immediate treatment unless they have symptomatic nodal disease, compromised end‐organ function, or B symptoms [9]. In our case, the patient was in an asymptomatic state and did not desire treatment. Therefore, treatment was not initiated, and we chose to monitor him closely.

In conclusion, although the frequency of recurrent follicular lymphoma together with diffuse granular shadow of the lung is uncommon, recurrent malignant lymphoma should be considered for differential diagnosis in patients with a history of malignant lymphoma.

### Disclosure Statement

Appropriate written informed consent was obtained for publication of this case report and accompanying images.

### Author Contribution Statement

Shotaro Okachi is the guarantor of the content of this manuscript. Takayasu Ito contributed to the draft of the manuscript. Yuichi Ishikawa, Satoko Shimada, Keiko Wakahara, and Naozumi Hashimoto contributed to the editing of the manuscript.
